# Circadian Mechanisms Underlying Reward-Related Neurophysiology and Synaptic Plasticity

**DOI:** 10.3389/fpsyt.2015.00187

**Published:** 2016-01-12

**Authors:** Puja K. Parekh, Colleen A. McClung

**Affiliations:** ^1^Department of Psychiatry, University of Pittsburgh School of Medicine, Pittsburgh, PA, USA

**Keywords:** circadian, synaptic transmission and plasticity, psychiatric disorders, mouse models, reward

## Abstract

Evidence from clinical and preclinical research provides an undeniable link between disruptions in the circadian clock and the development of psychiatric diseases, including mood and substance abuse disorders. The molecular clock, which controls daily patterns of physiological and behavioral activity in living organisms, when desynchronized, may exacerbate or precipitate symptoms of psychiatric illness. One of the outstanding questions remaining in this field is that of cause and effect in the relationship between circadian rhythm disruption and psychiatric disease. Focus has recently turned to uncovering the role of circadian proteins beyond the maintenance of homeostatic systems and outside of the suprachiasmatic nucleus (SCN), the master pacemaker region of the brain. In this regard, several groups, including our own, have sought to understand how circadian proteins regulate mechanisms of synaptic plasticity and neurotransmitter signaling in mesocorticolimbic brain regions, which are known to be critically involved in reward processing and mood. This regulation can come in the form of direct transcriptional control of genes central to mood and reward, including those associated with dopaminergic activity in the midbrain. It can also be seen at the circuit level through indirect connections of mesocorticolimbic regions with the SCN. Circadian misalignment paradigms as well as genetic models of circadian disruption have helped to elucidate some of the complex interactions between these systems and neural activity influencing behavior. In this review, we explore findings that link circadian protein function with synaptic adaptations underlying plasticity as it may contribute to the development of mood disorders and addiction. In light of recent advances in technology and sophisticated methods for molecular and circuit-level interrogation, we propose future directions aimed at teasing apart mechanisms through which the circadian system modulates mood and reward-related behavior.

## Introduction

Biological rhythms are ubiquitous throughout nature and play a pivotal role in helping organisms navigate dynamic environmental conditions to ensure survival and adaptive behaviors (1, 2). These complex systems have emerged as the result of evolutionary mechanisms allowing animals to physiologically and behaviorally entrain to a roughly 24-h day length (3). The basis for this entrainment ability lies in the molecular clock and the concerted function of several rhythmic tissues throughout the body, and particularly within the brain. In the mammalian brain, the suprachiasmatic nucleus (SCN) of the hypothalamus sets rhythms in response to light input through intrinsically photosensitive retinal ganglion cells (ipRGCs), which form the retinohypothalamic tract (4–6). The SCN communicates with subsidiary oscillators throughout the brain and in the periphery via peptide and neurotransmitter signaling to regulate physiology and behavior (7). Molecular clock machinery exists in all cells and is driven by transcriptional-translational feedback loops (TTFLs) in which the activity of individual components is regulated over a diurnal timescale. At the heart of the clock are circadian transcription factors Circadian Locomotor Output Cycles Kaput (CLOCK), the homologous protein Neuronal PAS Domain Protein 2 (NPAS2), and Brain and Muscle Arnt-like Protein 1 (BMAL1) with which CLOCK or NPAS2 heterodimerize to activate the transcription of Period (*Per1, Per2, Per3*) and Cryptochrome (*Cry1, Cry2*) genes. In the cytoplasm, PER and CRY proteins are translated and re-enter the nucleus to bind the CLOCK/NPAS2:BMAL1 transcriptional complex, inhibiting it and forming a negative feedback mechanism, which is completed over the course of 1 day (8–11). The clock is further stabilized by the nuclear receptors, *Rev-erb*α** and *Ror*α/β, which act to inhibit and activate the transcription of *Bmal1* and *Clock*, respectively, through their interaction with ROR elements. Several regulatory kinases, phosphatases, and accessory feedback loops complete the molecular clock adding additional complexity (12–15). Importantly, while participating in hierarchical feedback loops to maintain cellular rhythmicity, circadian transcription factors also regulate the expression of numerous other clock-controlled genes (CCGs). In fact, it is currently estimated that approximately 43% of the mammalian genome is rhythmic and these CCGs are involved in a wide array of physiological functions throughout the body and in the brain (16). In addition to the wide array of physiological and behavioral functions controlled by the circadian clock, the implications for circadian regulation of mood and reward behavior are beginning to be better understood at the molecular level.

## Circadian Rhythms and Psychiatric Illness

Circadian disruption appears to be both a symptom and a precipitating feature of some psychiatric conditions. For instance, the “social jet lag hypothesis” suggests that the weekly disturbances in sleep–wake rhythms imposed by work or school obligations, particularly in adolescence, correlate with an evening circadian typology as well as changes in overall well-being and stimulant consumption measured by self-report (17, 18). Sleep problems and seasonal variations in day/light hours can also cause some individuals to experience depression and can lead to self-medication through drug or alcohol abuse (17, 19, 20). Additionally, the “social zeitgeber hypothesis” proposes the idea that mood episodes may occur when disruptions in social routine are experienced, which lead to circadian rhythm and behavioral dysfunctions. Circadian rhythms in the use and sensitivity to several different classes of drugs have been observed as well (21–25). Genome-wide association studies (GWAS) link polymorphisms and other mutations in core circadian genes with seasonal affective disorder (SAD), major depression (MDD), addiction disorders, and bipolar disorder (BD), which is characterized by spontaneous mood cycling through depressive, euthymic, and manic phases (26–29). For a detailed description of known circadian mutations in humans associated with mood disorders, refer to an in-depth review by McCarthy and Welsh (30). A genetic basis for chronotype (preference for morning or evening consolidation of activity) in humans has been suggested by a number of studies as well, with “eveningness” a characteristic of BD in some patients (31–33). Pharmacological therapies for mood-related illnesses such as lithium and agomelatine, an antidepressant, may produce therapeutic effects through their stabilization of circadian rhythms, further highlighting the role of biological clocks in these disorders (34–39). Circadian gene mutation and chronotype can also correlate with an abnormal response to reward. A particular single nucleotide polymorphism (SNP) in the human *Period* gene, for instance, disrupts prefrontal reward responsivity and cortico-striatal activation following a rewarding stimulus (40, 41). These and other findings suggest an important role for circadian misalignment in the pathophysiology of mood and addiction disorders.

**Figure 1:**
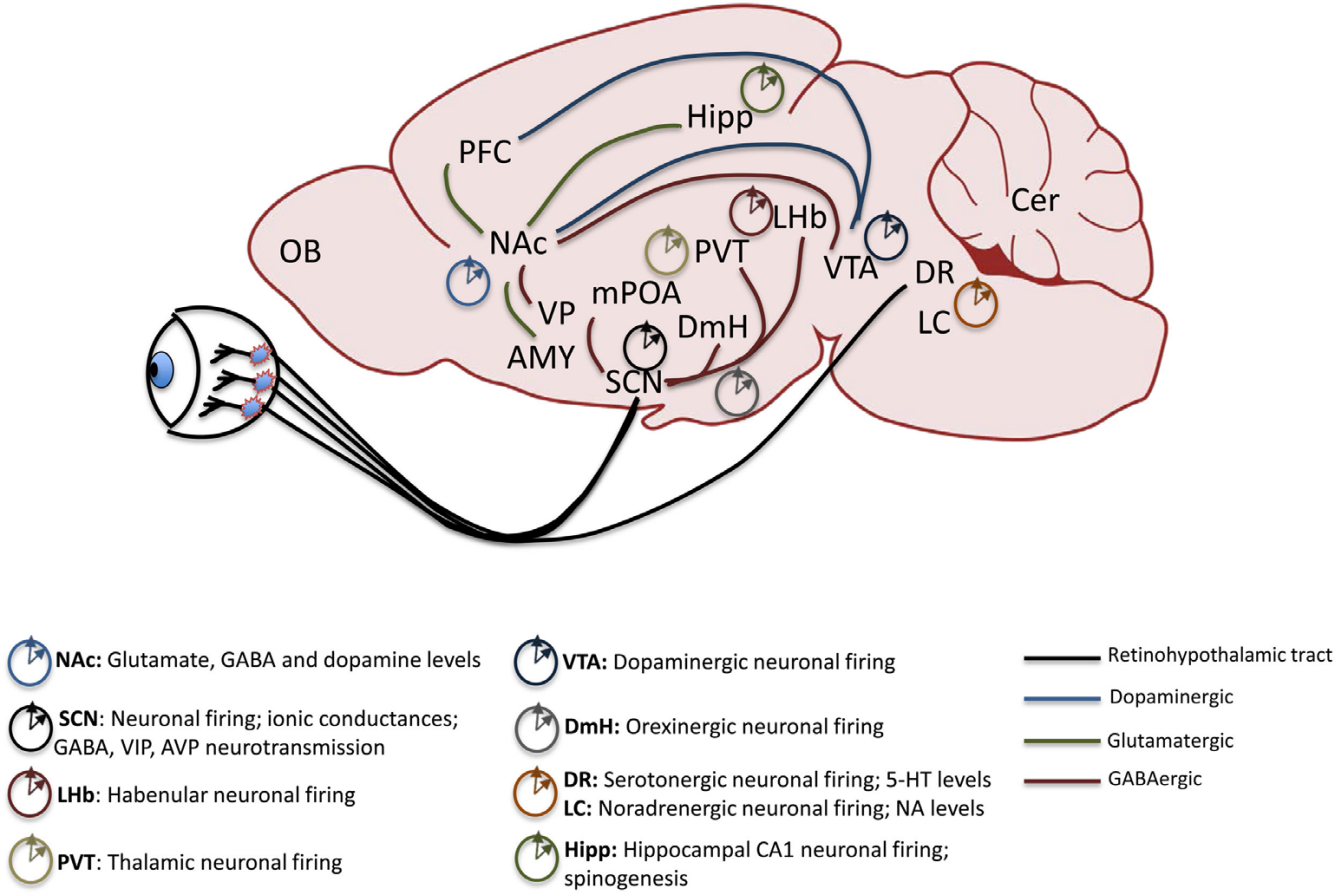
**Mesolimbic brain regions display rhythmic patterns in neural activity**. The retinohypothalamic tract provides light information and consists of ipRGC projections from the retina to the SCN and DR. The SCN provides direct GABAergic input to the LHb, DmH, mPOA, and the PVT. Reward-related pathways include a dopaminergic projection from the VTA to the NAc and PFC, glutamatergic inputs from the Hipp, Amy and PFC to the NAc and GABAergic output of the NAc to the VP and VTA. Known rhythmic components of neural activity in each of the mood and reward-related extra-SCN brain regions are denoted. ipRGC, intrinsically photosensitive retinal ganglion cells; SCN, suprachiasmatic nucleus; DR, dorsal raphe nuclei; LHb, lateral habenula; DmH, dorsomedial hypothalamus; mPOA, medial pre-optic area; PVT, paraventricular thalamic nucleus; VTA, ventral tegmental area; NAc, nucleus accumbens; PFC, prefrontal cortex; Hipp, hippocampus; Amy, amygdala; VP, ventral pallidum; LC, locus coeruleus; OB, olfactory bulb.

Work in pre-clinical animal models of psychiatric disorders highlights the central role of proper signaling in several key brain regions to maintain biochemical and neurophysiological balance within circuits. Reward circuitry is directly impinged upon by drugs of abuse and is also the main site of dysregulation in some mood disorders including BD (42–45). While direct projection targets of the SCN, including the medial pre-optic area (mPOA) and dorsomedial hypothalamic nucleus (DmH), are not central to the reward circuitry, they may modulate it through indirect neural connections (46). Orexinergic neurons in the DmH, for instance, encode information about arousal, energy balance, and reward, and project to the ventral tegmental area (VTA) a main source of dopamine (DA) in the brain (47–50). The dorsal raphe (DR) nuclei of the midbrain receive direct light input from the circadian visual system and also indirect input from the SCN and are the primary regions containing serotonin (5-HT) neurons in the brain. 5-HT is an important mood-related neurotransmitter (51). The lateral habenula (LHb) in the midbrain also receives direct SCN input and has been shown to be an important inhibitor of DAergic activity in the VTA, thus exerting a more robust influence over mood and reward regulation (52–54). In the traditional view of the reward system, projections between certain regions are highlighted as critical to the expression and maintenance of proper reward sensitivity and behavioral response. Among these are DAergic and GABAergic projections from the VTA and substantia nigra (SN) to the nucleus accumbens (NAc) and dorsal striatum (Str), respectively. The NAc is considered to be an integrator of sensorimotor information with limbic information to gate motivational behavior. Its extensive afferent and efferent connections serve to underscore this function. Chiefly, the NAc receives glutamatergic inputs from the PFC, amygdala (Amy), and hippocampus (Hipp) and provides GABAergic input to the VTA as well as the ventral pallidum (VP). The VTA also sends afferent inputs to the prefrontal cortex (PFC), a major site of executive function and cognitive control over behavior, and in humans, abnormal dopaminergic signaling within the PFC has been correlated with a drug-addicted state (42, 55). Each of these critical extra-SCN brain regions has been shown to maintain rhythms and to express circadian genes and proteins with clock and non-clock regulatory functions (see Figure 1). They therefore control mood and reward behavior through both circuit-level and molecular mechanisms (56–59).

## Circadian Rhythms in Neuronal Activity

In SCN neurons and other neuronal populations, the study of circadian regulation of ion channel mechanisms suggests further roles for these systems in neuronal excitability and signaling (11, 60, 61). The SCN is unique in its highly coupled network activity, which allows it to maintain robust endogenous rhythms in constant conditions by linking the molecular clock with machinery that controls cellular excitability (62–64). Insights into these mechanisms come from studies in many model organisms, including *Drosophila* melanogaster, which have several conserved molecular clock elements. Recent findings by Flourakis and colleagues have uncovered an ionic basis for diurnal rhythms in membrane excitability of central clock neurons, which are tied to the control of morning and evening activity patterns in both flies and rodents. Clock-controlled activity of both a resting sodium leak conductance and resting potassium conductance demonstrate an important link between molecular rhythms and membrane excitability across the light/dark cycle (65). Interestingly, while other studies have focused on circadian transcriptional regulation of ion channel genes themselves, this study finds that CLOCK rhythmically binds and activates transcription of a gene important for the proper axonal localization of a sodium channel that underlies leak conductance (65). This suggests that molecular oscillations can have diverse influences on cellular excitability through direct and indirect means.

Outside of the SCN as well, rhythms in neuronal activity have been observed. While it has long been thought that VTA DA neurons do not have a diurnal rhythm in firing rate, a recent study suggests that this may not be the case as an intra-diurnal rhythmic pattern of VTA DA neuronal activity has been measured in anesthetized rats (66). It is still unclear whether a strong link exists between firing and extracellular release of DA, however, in behaving animals. More work is needed to concretely establish these mechanisms. Additionally, the neuronal activity of the LHb and medial habenula (MHb) show rhythmic oscillation both *in vitro* and *in vivo*, and firing rates of neurons in both of these regions are altered in response to retinal illumination *in vivo*. The LHb maintains endogenous molecular rhythms as well with oscillations in *Per2* gene and protein levels across the light/dark cycle. Temporal variation in electrophysiological properties in each of these neuronal populations is absent in mice lacking a functional intracellular molecular clock. These findings support the idea that intrinsic circadian signals can shape the contribution of habenular nuclei to affective and reward behavior (30, 67, 68). Another reward-related region in the thalamus, the paraventricular nucleus (PVT), which sits at the midline and projects to many limbic structures including the NAc, also displays rhythms in activity. The PVT receives input from the SCN and the DmH and has been shown to play a role in the anticipatory locomotor response to food. The firing rate of PVT neurons varies throughout the day with greatest activity seen during the animal’s active phase (69–71). The influence of this small nucleus on reward sensitivity and drug seeking is beginning to be further elucidated (72, 73). Serotonergic neurotransmission, which is critically involved in the regulation of mood behavior, is significantly affected by photoperiod. Day length during development can alter firing properties of serotonin neurons as well as extracellular levels of 5-HT and norepinephrine. These light-induced changes further affect anxiety and depressive-like behavior (74–77). Work from the Aston-Jones lab has provided insights into the circadian regulation of activity of noradrenergic neurons in the locus coeruleus (LC), a key mediator of wakefulness and behavioral arousal. Using single-unit recordings of LC neurons in anesthetized rats, they have demonstrated that the neurons fire significantly faster during the active phase compared with rest phases. Additionally, the diurnal rhythm of noradrenergic neuronal activity correlates with the rhythm of activation of DmH orexin neurons, which project preferentially to the LC (78, 79). These and other studies highlight that important aspects of neuronal activity throughout the brain are under circadian influence and that the rhythmic activity of mood and reward-related regions may be relevant for behavioral outcomes.

## Circadian Rhythms are Involved in Synaptic Plasticity Mechanisms

A core mechanistic underpinning of many psychiatric illnesses is aberrant synaptic plasticity both within limbic regions and between cortical and subcortical areas (80–83). DA and glutamate neurotransmission have been implicated by several studies to be involved in the pathophysiology of BD, for instance, causing the disease to be framed by some as a synaptic plasticity-related disorder (84–86). Circadian rhythms in DA, glutamate and GABA levels in the dorsal striatum and NAc have been measured in awake rats, and these rhythms are independent of light in the NAc (87). Manic-like behavior can be modeled in rodents by the disruption of DA uptake mechanisms including dopamine transporter (DAT) pharmacological inhibition and genetic knockdown (88, 89). Additionally, imaging studies point to a decrease in DAT availability in the caudate of untreated BD patients (90). Along with decreased DAT transcript and protein levels in BD post-mortem cortical tissue and the correlation between DAT gene polymorphisms with predisposition to BD, there is strong evidence to support the role of DA homeostatic dysregulation in the disorder (90–93). Deficiency of decision-making seen in patients with BD is attributed in part to lower DAT functioning. This high-reward sensitivity and risk-preference can be measured in humans using the Iowa Gambling Task (IGT). Adaptation of the IGT to assess risk-based decision-making in mice reproduces human results in DAT-impaired conditions (88). Recent pre-clinical work points to a circadian regulation of DAT function in mice. Ferris and colleagues have demonstrated that diurnal variation in DAT activity accounts for rhythms in DA release in the striatum, and they have also shown diurnal oscillations in presynaptic D2 autoreceptor function (94). These findings could help explain how circadian dysregulation might contribute to the mood swings seen in BD patients. Interestingly, mood stabilizing pharmacological agents including valproate appear to have effects on both molecular clock components as well as DAT function and mRNA levels in rodents (95, 96). While many mood-stabilizers are not specific to any particular neurotransmitter system, it is worth noting that some of their targets include DAergic and glutamatergic pathways. DA receptor density changes are somewhat inconsistent; however, glutamatergic abnormalities are more clearly seen in BD patients. Findings have reported reduced levels of ionotropic glutamate receptor α-amino-3-hydroxy-5-methyl-4-isoxazoleproprionic acid (AMPAR) subunits in cortical areas of mood disorder subjects and reduced gene expression of the GluA1 subunit in striatal regions of BD patients (97–99). Additional proteins associated with the structural integrity of the post-synaptic density and proper trafficking of glutamate receptors to the membrane, including the scaffolding protein, PSD-95, and synapse-associated protein 102 (SAP102), have been found to be altered in post-mortem brains of BD patients (100, 101). These alterations may potentially lead to disruptions in excitatory signaling in mesocorticolimbic brain regions affecting mood and reward behavior. A GluA6 mutant mouse model of bipolar mania has also been characterized, as have pharmacological models including ketamine administration (102, 103). Additionally, polymorphisms in N-methyl-d-aspartate (NMDA) glutamate receptor genes correlate with susceptibility to BD (104, 105). Given the importance of DA-GLU interaction at postsynaptic sites for normal synaptic plasticity processes, it will be critical to follow up these findings with functional studies in disease models.

**Figure 2:**
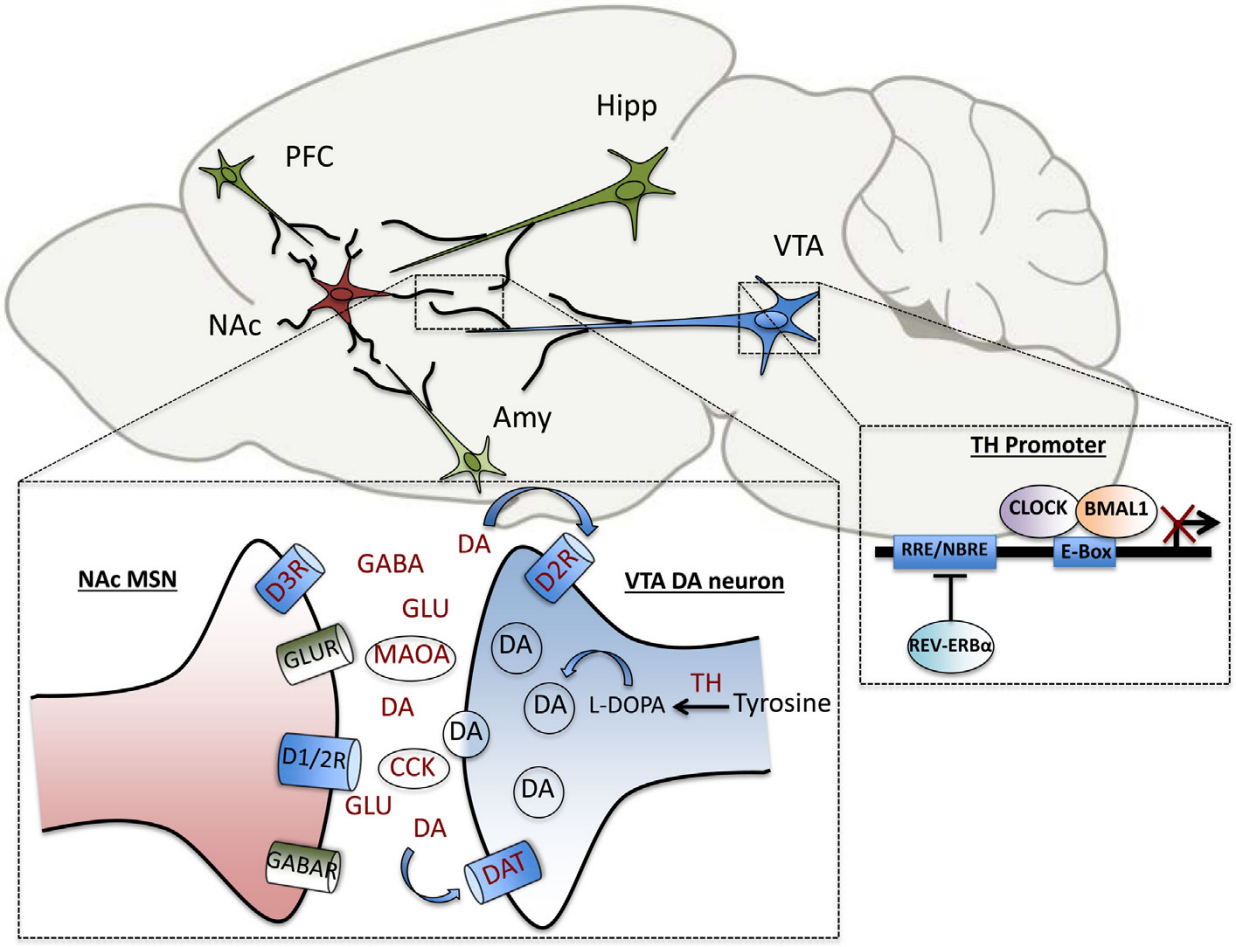
**Elements of dopaminergic transmission are under direct circadian control**. Within the VTA-NAc circuitry, clock genes regulate the transcription of several genes involved in the synthesis, uptake, transmission and degradation of dopamine, including tyrosine hydroxylase (TH), dopamine transporter (DAT), pre-synaptic dopamine type-2 receptor (D2R), dopamine type-3 receptor (D3R), monoamine oxidase A (MAOA), and cholecystokinin (CCK). Within the NAc, diurnal rhythms in levels of the neurotransmitters, dopamine, glutamate, and GABA have been measured as well. The transcription of TH is repressed by the circadian transcription factor, CLOCK, as well as the nuclear receptor, REV-ERBα, which bind to enhancer box (E-Box) and ROR response-element (RRE) sites in the promoter region, respectively.

Pre-clinical studies from our own group in a genetic mouse model of circadian disruption, the *Clock*Δ19 mice, have uncovered additional DAergic genes that are direct transcriptional targets of the core circadian transcription factor, CLOCK. These include tyrosine hydroxylase (*TH*), the rate-limiting enzyme in DA synthesis, and cholesystekinin (*Cck*), a peptide negatively associated with DA activity *in vivo*, which has been implicated in anxiety and drug response (106, 107). In addition to the regulation of DA synthesis and transmission, DA degradation has also been shown to be under circadian control. The clock proteins, PER1 and PER2 are rhythmically expressed in the striatum and *Per2* mutation disrupts the rhythmic activity of monoamine oxidase A (MAOA), the critical enzyme involved in DA catabolism (108). Recently, we have also demonstrated that CLOCK regulates the expression of *TH* by binding enhancer box (E-box) sequences at proximal and distal promoter regions of the gene. Known dopaminergic targets of circadian genes are summarized in Figure 2. In wild-type mice, CLOCK is a negative regulator of *TH* activation, and the dominant negative CLOCK protein in *Clock* mutant mice is therefore unable to repress *TH* transcript and protein levels, which are increased throughout the light/dark cycle. Consequently, extracellular DA levels are also increased in striatal regions in mutants (107). Behaviorally, the mutant mice show robust hypersensitivity to rewarding substances, reduced anxiety and depressive-like behavior and hyperactivity in response to novelty (109–111). The effect of these elevated DA levels on synaptic transmission and efficacy in target regions is being investigated on a functional level. Correlative evidence of circuit-level disruptions in *Clock*-mutant mice that may be the result of synaptic weight changes in cortico-limbic regions has also been demonstrated. Mutants display a decrease in cross-frequency phase coupling of low-gamma oscillations with delta oscillations within the accumbens corresponding to the time during which they explore open arms in an elevated zero maze (112). This suggests signaling deficits that may contribute to their reduced anxiety-like behavioral phenotype. These deficits may in part be due to altered excitatory drive onto accumbal medium spiny neurons as computational models suggest (112, 113). Interestingly, protein levels of the AMPA receptor subunit, GluA1, and phosphorylated GluA1, are decreased in the NAc of *Clock* mutants compared with wild-type littermates, indicating a post-synaptic adaptation that may be secondary to the increased DAergic tone in these animals (114). It will have to be determined whether the CLOCK protein directly regulates any aspects of glutamatergic signaling in this region.

Transcription factors are important in positive and negative regulation of plasticity-related genes and neuronal activity. The study of this regulation in other models sheds light on how these mechanisms might be occurring to control mood and reward behavior. For instance, social-defeat stress, a validated model of depression in mice, is thought to rely partly on the actions of cAMP response-element binding protein (CREB) to affect excitability of NAc medium spiny neurons, which are integral to affective and reward-related behavior (115). CREB signaling is increased in fibroblasts from BD patients as well (116). Another transcription factor, nuclear factor kappa B (NfκB), which is prominent in the striatum, is involved in reward processing and is increased following repeated cocaine administration (117, 118). Evidence for the positive regulation of NfκB-mediated transcription by CLOCK (independent of BMAL1 binding) indicates that circadian transcription factors can affect the activity of non-circadian transcription factors through protein interactions (119). Genes involved in accessory or stabilizing loops of the molecular clock machinery can also be important for behavior. It has recently been shown that the circadian nuclear receptor, REV-ERBα, which represses the transcription of *Bmal1*, has a key function in the direct regulation of *TH* expression in the ventral midbrain as well (120). By competing with the DA neuron-enriched nuclear receptor, NURR1, REV-ERBα is able to contribute to the rhythmicity of the DAergic system and affect mood behavior (120). This further suggests that clock proteins have a role in reward behavior outside of molecular clock functions.

## Rhythms in Learning and Structural Plasticity

The effects of circadian disruption on long lasting or homeostatic plasticity in reward and mood-related circuitry need to be further elucidated. Circadian rhythms in learning and memory processes help underscore the importance of these mechanisms for adaptive behavior requiring plasticity. Hippocampal-dependent learning in rodents as measured by the novel-object recognition task fluctuates over a diurnal timescale with performance peaking during the dark (active) phase (121, 122). In diurnal grass rats, long-term retention of reference memory in the Morris Water Maze is highest during the day, which is anti-phase to the performance of nocturnal rats (123). Many studies have found circadian oscillations of hippocampal signaling events in mice, and that these events are required for memory formation and adaptation to novel environments. *Bmal1* null mutant mice display impaired contextual fear and spatial memory as well as hyperactivity in novel environments and reduced habituation. Additionally, long-term potentiation (LTP) in hippocampal slices from these mutants is significantly decreased compared with wild-type mice (124, 125). *Npas2* mutant mice also show impairments in cued and contextual fear memory (126). Interestingly, daily changes have been demonstrated in the expression of genes encoding synaptic scaffolding proteins and also in the morphology of dendrites themselves. Circadian peaks in the stress hormone, corticosterone (CORT) enhance the formation of spines on pyramidal neurons in the motor cortex of rats (127–129). Functional effects of circadian oscillations on synaptic plasticity in the somatosensory cortex of mice have been examined in constant darkness conditions where the cyclic changes in the density of excitatory synapses on spines were abolished. These findings suggest that the density of excitatory synapses shows daily changes while the density of inhibitory synapses in this cortical region shows circadian changes (130, 131). Recent important work from the laboratory of Takao Hensch demonstrates that the *Clock* gene regulates critical period plasticity in the primary visual cortex of mice. They have found that CLOCK plays a role in the functional maturation of parvalbumin (PV) neuronal circuits, which comprise a primary source of inhibitory activity in the neocortex (132). A number of psychiatric conditions, including schizophrenia and autism, are characterized by abnormal excitatory/inhibitory balance in cortical circuits, and specifically, reduced functionality of PV interneurons; therefore, clock-mediated regulation of genes controlling synaptic and homeostatic events is of particular interest (133–135).

Outside of the retina and SCN, there are few studies that have systematically examined structural circadian remodeling of synapses in the brain. A small number of studies have demonstrated a circadian regulation of electrical activity in the olfactory bulb (OB) where the functional interaction of AMPARs with connexins to form gap junctions underlies the firing of action potentials. In this region, GluA1 mRNA and protein in particular were found to be strongly rhythmic across 24- and 48-h timescales. These synaptic interactions that govern the circadian synchronization of action potentials in the OB may impact olfactory coding and learning (136, 137). In zebrafish, a diurnal vertebrate model organism, a transparent body offers the unique advantage of visualizing structural plasticity. Synapses on hypocretin neurons important for sleep homeostasis show dynamic diurnal changes in live zebrafish. Rhythmic synaptic density in hypocretin axons is primarily regulated by the circadian clock potentially through transcriptional control of the expression of *nptx2b*, a gene that is important for AMPA receptor clustering (138, 139). Given the relevance of expression and function of synaptic proteins in disease states, the continued study of possible mechanistic influences of circadian clock elements in these processes will be critical.

## Rhythms in Neuroadaptations Associated with Drugs of Abuse

The investigation of neuroadaptations in reward-related brain regions following exposure to addictive substances, including methamphetamine, cocaine, morphine, and alcohol has been a mainstay of addiction research for decades. Many of these adaptations also involve the neuromodulatory effects of DA on glutamatergic and GABAergic synapses to re-wire circuits for abnormal reward learning (80, 140–142). Drugs of abuse can alter rhythmicity of core clock genes, and the activity of these genes can in turn affect the expression of proteins important for plasticity, suggesting a bidirectional relationship between the circadian and reward systems (143–146). Drugs provide potent non-photic entrainment cues affecting behavioral rhythms in a time-of-day dependent manner and potentially acting upon SCN electrical rhythms directly (23, 147, 148). Diurnal variations in amphetamine-induced locomotor activity, conditioned place preference for amphetamine, and the expression of *TH* mRNA in the VTA and NAc have been observed (149). Mutations of circadian genes, *Per1* and *Per2* in mice have led to opposing reward phenotypes where *mPer1* mutant mice display a lack of cocaine sensitization in response to repeated injections of the drug, while sensitization is robustly increased in *mPer2* mutants. Cocaine reward as assessed by place preference showed similar trends in the mutants, respectively. Cocaine-related reward behaviors in C57/BL6J mice are under circadian control and vary by zeitgeber time, highlighting the importance of taking into account diurnal differences in reward response (150). Abnormally increased alcohol consumption in *Per2*^(Brdm1)^-mutant mice is associated with altered glutamatergic activity where lower levels of the glutamate transporter gene, *Eaat1*, lead to increased extracellular levels of glutamate in the brain. Furthermore, acamprosate, a therapeutic agent used to prevent craving and relapse in alcoholic patients, is effective in normalizing glutamate levels and alcohol consumption in mutant mice (151).

Our group has characterized reward-related dysfunction in *Clock*Δ19 mice including a robust sensitization to cocaine, increased preference for cocaine, and increased goal-directed behavior, and motivation in measures of cocaine self-administration. Additionally, these mice exhibit higher motivation for self-stimulation of the medial forebrain bundle and preference for sucrose and alcohol (109–111). Interestingly, while *Npas2* is thought to be a paralog of *Clock* with high structural and functional homology, *Npas2*-mutant mice show a decrease in cocaine-conditioned place preference, a measure of reward sensitivity (146, 152, 153). This discrepancy in the role of the two circadian transcription factors in mediating reward response could be due to differential expression of the genes in the brain. *Clock* is ubiquitously expressed throughout the brain, while *Npas2* is highly enriched in the forebrain, including specifically the NAc, and is not expressed in the VTA where *Clock* is known to be critical for reward behavior (126). Additionally, viral-mediated knockdown of NPAS2 within the NAc itself is sufficient to decrease cocaine preference in C57/BL6 mice. In this brain region, NPAS2 also directly regulates the expression of the DA D3 receptor by binding to a non-canonical E-box sequence in the promoter region of the *Drd3* gene. Chronic cocaine administration also increases the expression of *Npas2* in the striatum where the gene has been shown to be highly enriched in the D1 DA receptor containing medium spiny neurons (146). The two main subpopulations of MSNs, D1 receptor- and D2 receptor-containing, are differentially involved in reward regulation due to the opposing actions of the receptor types on cellular activity via G-protein signaling mechanisms, and the projection pathways of neurons. D3 receptors have been proposed to play a role in reward seeking potentially through membrane interactions with D1 receptors (154–157). The myriad ways in which clock proteins directly and indirectly mediate drug response potentially through the control of synaptic proteins will have to be investigated further.

## Future Directions

As we continue to piece together the mechanisms by which the circadian system regulates neuronal activity, synaptic plasticity, and behavior, it will be valuable to take stock of the variety of techniques available for further interrogation as well as the many outstanding questions. We have uncovered aspects of clock function in core limbic circuitry; however, other brain regions will need to be investigated as well since we know that psychiatric conditions involve disruptions in many mesocorticolimbic structures and pathways. Addiction, for instance, is believed to progress through cascades of dysfunction affecting several brain regions (158). Additionally, the study of other neurotransmitter systems beyond DA in circadian gene models of mood and reward disorders will be important. Mood cycling in BD has been proposed to involve neurotransmitter switching, for instance (159). Recent advances in technology for interrogating circuitry include optogenetics and pharmacogenetics. Beyond the use of traditional opsins that alter neuronal excitability by depolarizing and hyperpolarizing cell membranes (channelrhodopsin and halorhodopsin) a variety of other, sophisticated constructs have been designed to target cellular mechanisms including G-protein signaling (Opto XRs) and designer receptors activated by designer drugs (DREADDs) (160, 161). These tools can be applied in the ongoing effort to understand how direct and indirect SCN projections to mesocorticolimbic areas affect behavior in a regionally and temporally specific manner. The contribution of subsidiary oscillatory networks in extra-SCN regions to behavior can be assessed further by anatomical mapping and pathway-specific manipulations.

As noted by our group and others, clock genes can exert differential effects in extra-SCN brain regions that may have diverse or opposing influences on behavior. The differences in expression of these genes and proteins though they may have overlapping roles in the SCN are important to consider, as the downstream effectors may be cell-type or circuit-specific. Microarray analysis and Chromatin-immunoprecipitation followed by deep sequencing have allowed us to identify several target genes that are bound by circadian transcription factors in normal or altered physiological conditions. These genes can be categorized by their cellular functions and profiles can be made of binding activity across diurnal time scales. Following up these large-scale analyses with reporter gene assays to determine transcriptional activity of circadian proteins is also critical to understand their function. These methods have proven very useful in the identification of clock gene targets that regulate DAergic transmission and post-synaptic activity in the VTA-Str circuitry. Many other genes relevant to synaptic plasticity known to have rhythmic activity can be explored, as some of their protein products are targets of pharmacotherapeutic agents. One promising new advance involves the optical control of transcriptional effectors by custom constructs utilizing cryptochrome proteins. While this TALE-LITE system has primarily been validated *in vitro*, it holds the potential to be useful for the precise manipulation of circadian transcription factors in key brain regions (162). Altering the rhythmicity of these proteins may provide insights into how their regular function affects cellular and synaptic activity and the consequences of circadian misalignment on these functions. The benefits provided by these approaches extend beyond the traditional use of genetic mutant models and viral-mediated gene transfer though these have proved extremely useful in first characterizations of the effects of circadian disruption on behavior.

While animal models are very valuable, a variety of cell models can be put to use to gain mechanistic information and for the direct translational potential of studying human patient populations. Blood biomarkers, inducible pluripotent stem cells (iPSCs) and skin fibroblasts for instance have yielded a wealth of information about cellular abnormalities in disease states and point to possible further diagnostic measurements (30, 163). Lastly, work over many decades has helped to characterize the electrophysiological properties of SCN neurons and the signaling networks that exist within the nucleus to orchestrate molecular rhythms. However, these same methods can be applied in mesocorticolimbic areas to uncover rhythms in cellular and network activity relevant to behavior. It will be important to continue to directly measure synaptic function at key microcircuits in circadian-mutant models with abnormal mood or reward-related phenotypes, or following circadian misalignment paradigms such as photoperiod manipulation or “shift-work” simulation. Given the regulatory role of clock proteins in activating or repressing transcription of CCGs and the relevance of synaptic plasticity to normal behavior and psychiatric illness, more work is needed to understand the intersection of these systems.

## Author Contributions

PP conceptualized and wrote the manuscript. CM provided editorial comments for the manuscript.

## Conflict of Interest Statement

The authors declare that the research was conducted in the absence of any commercial or financial relationships that could be construed as a potential conflict of interest.
